# A Sheep Identification Method Based on Three-Dimensional Sheep Face Reconstruction and Feature Point Matching

**DOI:** 10.3390/ani14131923

**Published:** 2024-06-29

**Authors:** Jing Xue, Zhanfeng Hou, Chuanzhong Xuan, Yanhua Ma, Quan Sun, Xiwen Zhang, Liang Zhong

**Affiliations:** 1 College of Mechanical and Electrical Engineering, Inner Mongolia Agricultural University, Hohhot 010018, China; jingjingtuzi@126.com (J.X.); njau-hzf@163.com (Z.H.); xcz@imau.edu.cn (C.X.); yanhuama@126.com (Y.M.); 2021202050031@emails.imau.edu.cn (Q.S.); 2 Inner Mongolia Engineering Research Center for Intelligent Facilities in Prataculture and Livestock Breeding, Hohhot 010018, China; 3 School of Computer and Information Engineering, Xiamen University of Technology, Xiamen 361024, China

**Keywords:** sheep identity recognition, deep learning, three-dimensional modeling, feature point matching, MobileViT, attention module

## Abstract

**Simple Summary:**

Accurate identification of individual sheep holds significant practical implications for modern sheep farming. Scholars have pioneered sheep face recognition technology based on deep learning techniques, enabling accurate identification of individual sheep through facial images. However, the existing sheep face recognition technology suffers from a limitation of a single identification method, where related studies solely rely on learning from sheep facial images to accomplish identification tasks. Therefore, this study proposes an innovative sheep face recognition approach that utilizes three-dimensional sheep facial reconstruction technology and feature point-matching models to achieve efficient identification of individual sheep. By developing novel identification methods, the aim is to surpass the limitations of solely image-based recognition and offer a wider range of options for sheep face recognition technology.

**Abstract:**

As the sheep industry rapidly moves towards modernization, digitization, and intelligence, there is a need to build breeding farms integrated with big data. By collecting individual information on sheep, precision breeding can be conducted to improve breeding efficiency, reduce costs, and promote healthy breeding practices. In this context, the accurate identification of individual sheep is essential for establishing digitized sheep farms and precision animal husbandry. Currently, scholars utilize deep learning technology to construct recognition models, learning the biological features of sheep faces to achieve accurate identification. However, existing research methods are limited to pattern recognition at the image level, leading to a lack of diversity in recognition methods. Therefore, this study focuses on the small-tailed Han sheep and develops a sheep face recognition method based on three-dimensional reconstruction technology and feature point matching, aiming to enrich the theoretical research of sheep face recognition technology. The specific recognition approach is as follows: full-angle sheep face images of experimental sheep are collected, and corresponding three-dimensional sheep face models are generated using three-dimensional reconstruction technology, further obtaining three-dimensional sheep face images from three different perspectives. Additionally, this study developed a sheep face orientation recognition algorithm called the sheep face orientation recognition algorithm (SFORA). The SFORA incorporates the ECA mechanism to further enhance recognition performance. Ultimately, the SFORA has a model size of only 5.3 MB, with accuracy and F1 score reaching 99.6% and 99.5%, respectively. During the recognition task, the SFORA is first used for sheep face orientation recognition, followed by matching the recognition image with the corresponding three-dimensional sheep face image based on the established SuperGlue feature-matching algorithm, ultimately outputting the recognition result. Experimental results indicate that when the confidence threshold is set to 0.4, SuperGlue achieves the best matching performance, with matching accuracies for the front, left, and right faces reaching 96.0%, 94.2%, and 96.3%, respectively. This study enriches the theoretical research on sheep face recognition technology and provides technical support.

## 1. Introduction

With the rapid development of modernization, digitization, and automation in animal husbandry, there is increasing attention towards precision and intelligent farming methods, necessitating the establishment of farms integrated with big data [1,2]. Therefore, in modern sheep farm management, there is a need to collect diverse individual information about sheep. These data aid farmers in devising precise breeding strategies to enhance efficiency, reduce costs, and promote healthy farming practices. Against this backdrop, the accurate and rapid identification of individual sheep becomes a prerequisite for collecting individual information [3].

Currently, most domestic breeding farms employ traditional methods for sheep identification, including ear tags, tattoo recognition, paint markings, wearing leg bands, and manual observation [4]. However, these traditional sheep identification methods suffer from low identification efficiency, significant identification errors, high labor requirements, frequent maintenance needs, and potential stress on the sheep. Consequently, these methods are not ideal for large-scale sheep herd management. Moreover, there is a risk of theft associated with using traditional sheep identification methods, as thieves can easily steal sheep by replacing ear tags, erasing marks, or removing leg bands, resulting in losses. Therefore, traditional sheep identification methods have significant limitations [5].

In modern sheep identification methods, some farms utilize radio-frequency identification (RFID) technology for sheep identification [6]. RFID technology employs radio-frequency ear tags to wirelessly identify and store the sheep’s identity information. Each sheep wears an ear tag containing a unique identification number, which can be wirelessly communicated with using a reader/writer to identify and record each sheep’s identity information [7,8]. Compared to traditional sheep identification methods, RFID technology is more accurate and efficient, reducing labor requirements and the possibility of error in recording [9]. However, RFID-based sheep identification methods face challenges such as high equipment costs and sensitivity to local signal-to-noise ratio. In complex farming environments, RFID technology may experience instability, further affecting the accuracy and reliability of identification [10]. Additionally, sheep activity in complex environments can lead to RFID ear tag wear, loss, or damage, necessitating additional costs and work for replacement or repair.

In recent years, scholars have begun leveraging deep learning techniques to study the biological features of livestock, achieving accurate identification of livestock identities [11,12]. Sheep face recognition technology, a biometric identification method, offers a low-cost, non-invasive, reliable, and efficient method for identity recognition [13,14]. Due to the rich biological features of sheep faces, different sheep exhibit high facial distinctiveness [15]. Moreover, among various livestock identification methods, facial recognition methods do not require direct contact with the animals, thereby achieving efficient identification while effectively avoiding stress behaviors in livestock [16,17,18]. Therefore, sheep face recognition technology is considered one of the most promising approaches for sheep identity recognition [19,20].

Song et al. [21] conducted facial data collection on 20 adult Sunite sheep using handheld video cameras during experiments. The collection process proceeded as follows. Each experimental sheep was individually brought into a closed pen and filmed while remaining calm. The enclosed pen used for housing the experimental sheep was rectangular, measuring 5 m in length and 3 m in width. Facial data of the sheep were captured in the form of video streams, recorded at a frame rate of 30 frames per second and a resolution of 1920 × 1080 pixels. Different angles and lighting conditions were employed for the facial filming of each experimental sheep to enhance the complexity and diversity of the collected data. Ultimately, an optimized YOLOv3 model was trained, achieving an mAP@0.5 of 96.8%.

Li et al. [22] developed a lightweight sheep facial recognition model called SheepFaceNet, achieving a balance between recognition speed and accuracy. The SheepFaceNet model comprises two key modules: SheepFaceNetDet for sheep face detection tasks and SheepFaceNetRec for sheep face recognition tasks. SheepFaceNet utilizes the Eblock module, which efficiently extracts sheep facial features. In SheepFaceNetDet, the backbone network is constructed using Eblock and incorporated into bidirectional FPN layers, enabling accurate sheep face detection. SheepFaceNetRec constructs a feature extraction network using Eblock and employs the ECA mechanism to enhance feature extraction, facilitating sheep face recognition. A diverse collection of sheep facial data was gathered in various environments, including indoor and outdoor settings and different lighting conditions, distances, and heights, resulting in 95 sheep facial videos comprising 78 alpine goats and 61 Tan sheep, with a video resolution of 1920 × 1080 pixels. After processing, a total of 7328 sheep facial images were obtained. Further, a self-constructed sheep facial image dataset was built after data augmentation. On the self-constructed sheep facial image dataset, SheepFaceNetDet achieves a recognition rate of 96.4%, identifying 387 images per second. Meanwhile, SheepFaceNetRec achieves a recognition accuracy of 97.8%.

To address the issues of low accuracy and slow speed in alpine goat facial recognition, Zhang et al. [23] proposed an improved YOLOv4 goat face recognition model named YOLOv4 goat-face-recognition. The study focused on alpine goats, with a total of 30 goats used for experimentation, resulting in the collection of 2522 goat facial images, further divided into training, validation, and testing sets. In the recognition model, the backbone network was replaced with a lightweight GhostNet feature extraction network. By optimizing the pyramid network and the path aggregation network, the model’s ability to distinguish facial features was further enhanced. During the training phase, transfer learning pretraining was employed to further improve the performance of the recognition model. In performing the goat face recognition task, the recognition model achieved an mAP@0.5 of 96.7%. Compared to YOLOv4, there was a 2.1% improvement in mAP@0.5. Additionally, when identifying alpine goat profiles, the recognition model achieved an mAP@0.5 of 78.0%, representing a 7.0% improvement compared to YOLOv4. The results demonstrate the excellent performance of the recognition model in goat facial recognition tasks.

While significant progress has been made in related research, current studies on sheep face recognition predominantly rely on pattern recognition techniques, wherein recognition models learn specific facial features from images to accomplish identity recognition. Consequently, the methods for sheep face recognition remain relatively singular. In comparison to facial recognition technology, current sheep face recognition methods primarily rely on image recognition, overlooking the potential advantages of other recognition approaches. Facial recognition methods encompass not only image recognition but also feature point matching and three-dimensional facial reconstruction, which, through deep learning techniques, can significantly enhance recognition accuracy and robustness. Therefore, there is a need for further research and development of diversified recognition methods in the field of sheep face recognition to fully exploit the advantages of different recognition approaches and improve the comprehensiveness and reliability of sheep face recognition.

This study focuses on the small-tailed Han sheep and proposes an innovative sheep face recognition method aimed at enriching the theoretical research of sheep face recognition technology. Firstly, utilizing three-dimensional reconstruction technology, three-dimensional facial images with prominent three-dimensional features are generated for each experimental sheep, including frontal, left-side, and right-side views. These generated three-dimensional sheep facial images only retain the facial region to eliminate interference from the background. Subsequently, a feature point-matching model is constructed using deep learning technology. When performing the matching task, this model matches the identification images with the generated three-dimensional sheep facial images. Finally, the sheep with the most matched points is selected as the final recognition result. By developing the aforementioned recognition method, the aim is to overcome the limitations of relying solely on image recognition and provide more diverse options for sheep face recognition technology.

The main contributions of this study are as follows.

A novel sheep identification method is proposed by combining three-dimensional modeling technology with feature point-matching algorithms.

An algorithm for sheep face orientation recognition, named SFORA, is introduced.

The SuperGlue algorithm is employed for the matching task, and experimental results suggest optimal performance when the confidence threshold is set to 0.4.

A sheep identification device is designed and developed to enhance identification efficiency, meeting the practical requirements of applications in livestock farming.

## 2. Material and Methods

### 2.1. Technical Route

**Figure 1:**
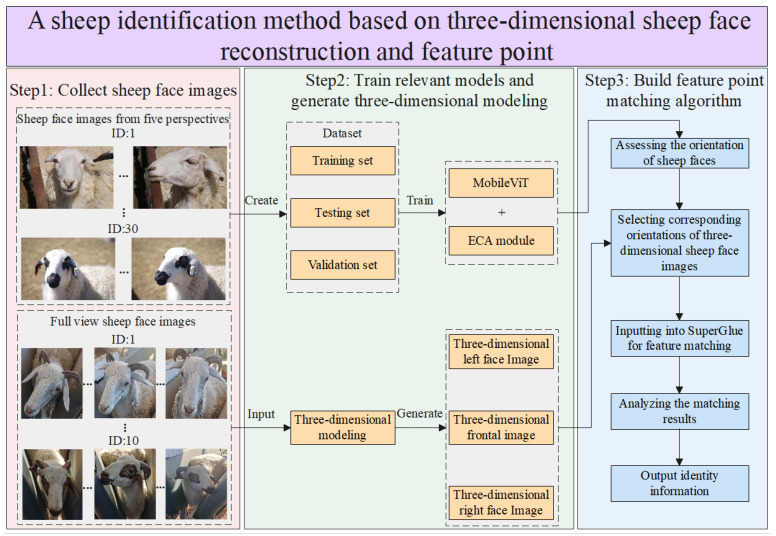
The workflow of the sheep face recognition method is based on feature point matching.

This study proposes a novel sheep face recognition method that requires a combination of multiple modules to accomplish a recognition task. The specific recognition process is as follows. Firstly, sheep face images are collected, which are categorized into two types: five perspectives of sheep face images and full-view sheep face images. The five perspectives of sheep face images are utilized for subsequent training of the sheep face orientation recognition algorithm, while the panoramic sheep face images are used for generating three-dimensional sheep face models. Through three-dimensional sheep face reconstruction technology, three-dimensional sheep face models of the target sheep are generated, further providing frontal, left-profile, and right-profile sheep face images. These three-dimensional sheep face images eliminate background interference while containing rich sheep facial feature information. When applying sheep face recognition technology in a farming environment, the orientation of the sheep face recognition images captured by the identification camera needs to be determined for subsequent effective matching. Moreover, this study constructs the SFORA based on MobileViT and ECA modules. The recognition images are processed by the SFORA to obtain their corresponding sheep face orientation output results, which are then matched with the stored three-dimensional sheep face images based on the subsequent feature-matching algorithm. The matching process involves three-dimensional sheep face images of all the sheep to be identified, and the sheep with the best matching effect is selected as the identity output for recognition targets. The workflow of the sheep face recognition method based on feature point matching is illustrated in Figure 1.

### 2.2. Sheep Face Orientation Recognition Algorithm

#### 2.2.1. Multi-Perspective Sheep Face Image Acquisition

The small-tailed Han sheep, a subspecies of the Mongolian sheep, is characterized mainly by white fur, with some individuals having black or brown spots on their faces. These spots are typically distributed around the eyes, ear tips, cheeks, or mouth. Due to its strong adaptability and stable breed characteristics, the small-tailed Han sheep is widely used in the sheep meat farming industry. In this study, a set of facial images of small-tailed Han sheep was collected using manual photography. The data collection took place in April 2022 at the breeding farm of Inner Mongolia Beiqi Technology Co., Ltd. (Hohhot, China), involving 30 experimental sheep. The experimental sheep used in this study were all sourced from the farm of Inner Mongolia Beiqi Technology Co., Ltd. The farm is equipped with professional veterinarians and staff who manage the sheep through intensive management practices. The age of each experimental sheep was controlled to be between one and three years old, and the collection period lasted approximately half a month.

Considering that sheep frequently move their heads in their daily activities, situations arise where they may not directly face the recognition camera during facial recognition. To address the above issue, this study collected multi-angle sheep face images to create a dataset. During the image collection process, sheep face images were captured from various angles and specifically categorized into five types: frontal, left half-face, left profile, right half-face, and right profile. The frontal images were taken with the camera directly facing the sheep’s face. Left half-face images focused on capturing facial details when the sheep’s head is tilted to the left, with the angle controlled between 30° and 60° to the left side. Left profile images were specifically collected to capture features of the left side of the sheep’s face, taken with the camera facing directly toward the left side of the sheep’s face. Similarly, the right half-face and right profile images corresponded to the right side of the sheep’s face. This collection strategy ensured that the dataset contained a comprehensive range of sheep facial features.

During the image collection process, selected experimental sheep were gathered in a pen and allowed to move freely. To ensure that the experimental sheep were not disturbed during the collection process, the photographer maintained a certain distance from the sheep. The photographer handheld the collection device to capture images of sheep faces. The pen was rectangular, measuring 20 m in length and 10 m in width, with a rain shelter installed along the perimeter. Specific shooting details are as follows. Sheep face images were captured using a single-lens reflex camera (Canon EOS 600D) and saved in JPG format with an image resolution of 2736 × 1824 pixels. The shooting distance from the sheep was controlled within 3 m to avoid capturing images from too far away, which could result in small facial regions or decreased resolution. To prevent high similarity between captured sheep face images, no automatic or continuous shooting operations were selected during the shooting process. The interval between capturing two sheep face photos was greater than 10 s.

**Figure 2:**
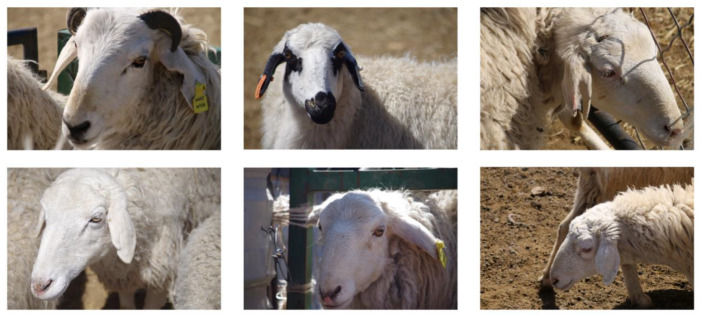
The sheep face image samples collected in this study.

Deep learning models can automatically learn features from datasets. Therefore, recognition models need to overcome challenges from diverse collection conditions in datasets to achieve higher recognition accuracy and robustness. When capturing sheep face images, random shooting distances and backgrounds were used to increase the diversity and comprehensiveness of the dataset. Additionally, the lighting conditions during shooting were natural indoor and outdoor lighting. The shooting time was divided into two parts: from 9 a.m. to 11 a.m. and from 2 p.m. to 6 p.m. Each experimental sheep was photographed with 150 facial images, including 30 images for each of the five angle groups. After manual inspection to remove blurry and incomplete facial images, each experimental sheep retained 120 facial images. The sheep face image samples collected in this study are shown in Figure 2.

#### 2.2.2. Sheep Face Orientation Dataset

During the process of sheep face recognition, various interference factors exist due to the complex environment of the breeding farm, such as lighting conditions, dust in the air, and changes in the posture of sheep heads. These factors pose challenges to the accuracy, effectiveness, and robustness of the recognition model. To address these challenges, various data augmentation methods were applied to the originally collected sheep face images in this study. Through multiple data augmentation methods, the aim was to enrich the diversity of sheep face images, which helps the recognition model adapt better to various recognition scenarios and improves its performance in practical applications. The data augmentation methods employed in this study included adjusting image brightness (−60% to 160%), adjusting image contrast (−50% to 160%), random rotation (−45° to 45°), adding Gaussian noise, horizontal flipping, and adding salt-and-pepper noise.

During the data augmentation process, this study employed a random approach where the frequency of each data augmentation method’s usage was randomized, ensuring that the number of sheep face images was quadrupled. A total of 14,400 augmented multi-angle sheep face images were obtained from the 30 experimental sheep, which were used to construct the sheep face orientation dataset. Subsequently, all sheep face images were divided into training, validation, and test sets in an 8:1:1 ratio. The specific configuration of the sheep face orientation dataset is presented in Table 1.

**Figure 3:**
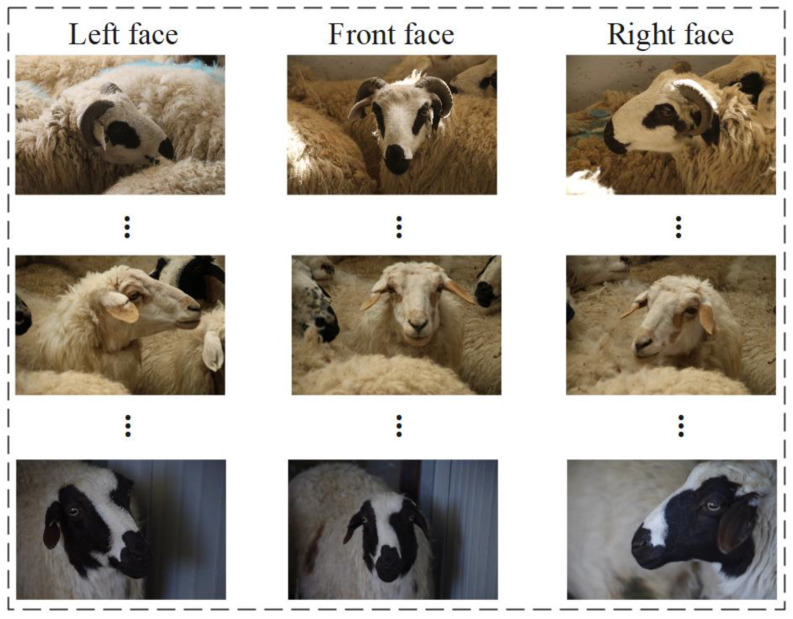
Schematic diagram illustrating the division of three sheep face orientations.

When creating a sheep face orientation dataset, it is necessary to divide the images in the dataset in detail. Specifically, the dataset was divided into three folders, with folder names labeled “Front face,” “Left face,” and “Right face.” Among these, the collected left half-face and left-profile facial images are defined as left face and right half-face, right profile facial images are defined as right face, and frontal facial images are defined as frontal face. In this study, we divided multi-view sheep face images into three groups of folders for subsequent model training. A schematic diagram illustrating the division of three sheep face orientations is shown in Figure 3.

#### 2.2.3. MobileViT

**Figure 4:**
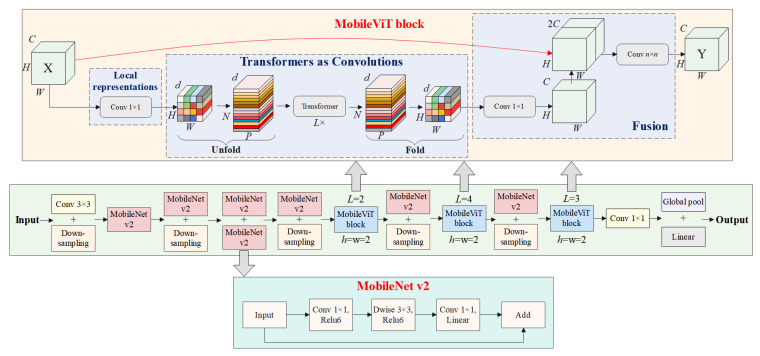
The overall structure of MobileViT.

MobileViT is a lightweight recognition model based on the transformer architecture, designed to achieve efficient image recognition and computation on mobile devices [24,25]. It combines the efficient feature extraction capability of CNNs with the global perspective provided by transformer’s self-attention mechanism [26]. MobileViT consists of ordinary convolutions, MobileNetV2 (MV2) modules, MobileViT blocks, global pooling, and fully connected layers [27]. The overall structure of MobileViT is illustrated in Figure 4.

MobileViT first undergoes a regular convolutional structure to extract the initial features of the input image. Then, it undergoes multiple sets of MV2 modules and MobileViT blocks for deep target feature extraction and learning. Finally, a global pooling layer pools the output features, and the resulting feature vector is entered into the fully connected layer for category prediction.

MobileViT consists of two key feature extraction modules, namely, the MV2 module and the MobileViT block. In the MV2 structure, the primary building unit is the inverted residual block, which is contrary to the traditional residual block. The inverted residual block first reduces the number of channels of the input data using lightweight 1 × 1 convolutional layers to achieve compression, then applies depth-wise separable convolutional layers for feature extraction, and finally expands the channels through 1 × 1 convolutional layers to achieve dilation. This design structure can increase the depth and nonlinear representation capability of the network without increasing computational complexity. Additionally, MV2 introduces a linear bottleneck structure, which further improves the model’s performance and efficiency by reorganizing the activation functions and linear operations in the expansion stage of residual blocks.

The purpose of the MobileViT block is to model global and local information with fewer parameters. As shown in Figure 4, the MobileViT block first conducts local feature extraction using a convolutional layer with an *n* × *n* kernel size, followed by channel adjustment using a 1 × 1 convolutional layer. Then, the output features are sequentially processed using the unfold, transformer, and fold modules for global feature modeling. “Unfold” unfolds the input feature map of size *H* × *W* × *d* into *P* × *N* × *d*, applies transformer for encoding, then uses “fold” to concatenate the encoded *P* × *N* × *d* feature map back to the original shape of *H* × *W* × *d*. Subsequently, a 1 × 1 convolutional layer adjusts the channels to the original size, and the output is concatenated with the original input feature map via a shortcut branch by channel concatenation. Finally, a convolutional layer of *n* × *n* kernel size is used for feature fusion, resulting in the final output.

The transformer module in the MobileViT block is used to extract high-level semantic features from input images. In MobileViT, the transformer module takes the feature map extracted by the convolutional layer as input, where each position’s feature vector represents the semantic information of the pixel at that position in the feature space. The transformer module utilizes a self-attention mechanism to encode these feature vectors, preserving local features while retaining global information, and then applies a feed-forward network to nonlinearly transform the features. The self-attention mechanism and the feed-forward network module work together to improve the model’s prediction accuracy.

In summary, MobileViT is a lightweight image recognition model with efficient computational performance. Considering the relatively low difficulty of the sheep face orientation recognition task in this section, a lightweight model is adopted as the sheep face orientation recognition algorithm to achieve efficient orientation determination. In this study, the lightest variant of the MobileViT series, MobileViT-XXS, is utilized as the sheep face orientation recognition algorithm.

#### 2.2.4. ECA Module

**Figure 5:**
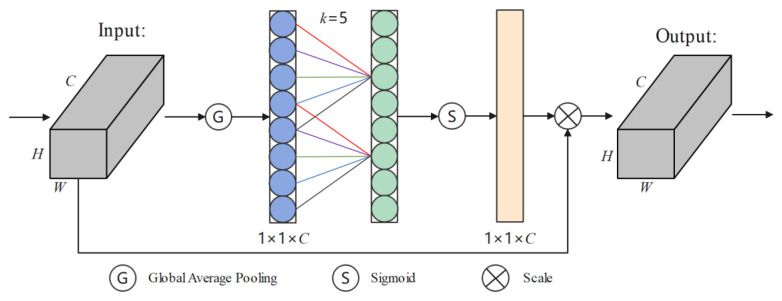
Structure diagram of the ECA mechanism.

ECA is a commonly used channel attention mechanism designed to improve the efficiency of model utilization of channel information while reducing computational complexity [28,29]. The ECA mechanism mainly focuses on how to more efficiently utilize the information in each channel of the convolutional layer. Compared to the SE attention mechanism, ECA adopts a more lightweight approach to channel attention [30]. The structure diagram of the ECA mechanism is shown in Figure 5.

The specific calculation process of the ECA mechanism is as follows: assuming the input feature map of the convolutional layer is *X*, with dimensions *C* × *H* × *W*, where *C* is the number of channels, *H* is the height, and *W* is the width. Similar to the SE attention mechanism, the input features *X* first undergo global average pooling, where the average of feature values within each channel is computed, resulting in a vector of length *C*. Subsequently, a convolution is applied to the vector *C*, where the adaptive convolution kernel *k* needs to be computed according to the following formula:

(1) $$k=\left|\frac{\log_{2}(C)}{\gamma }+\frac{b}{\gamma }\right|$$

where the value of *b* is 1 and the value of *γ* is 2 in this study.

The value of the adaptive convolution kernel *k* is set to 5 through computation. Therefore, a one-dimensional convolution with a kernel size of 5 is used to calculate the weights of the channels, followed by normalization through a sigmoid activation function, resulting in weights ω equal to the number of input channels. The calculation formula is as follows:

(2) $$\omega =\sigma (D_{k}\left(X)\right)$$

where *D_k_* denotes a one-dimensional convolution with a kernel of *k* and $\sigma \left(\cdot \right)$ denotes passing through the sigmoid activation function.

The obtained weight vector undergoes a scale operation and is multiplied by the original feature map to reinforce important channel information and diminish the impact of unimportant channels. Finally, the output feature Y is obtained, with the calculation formula as follows:

(3) Y=X×ω

Compared to other channel attention mechanisms, ECA is lighter in weight and has lower computational costs, making it suitable for recognition tasks with limited computational costs.

#### 2.2.5. Sheep Face Orientation Recognition Algorithm

To further improve the accuracy of sheep face orientation recognition, this study introduced ECA modules based on MobileViT-XXS. Specifically, ECA modules were introduced before the first MobileViT block and the second MobileViT block.

**Figure 6:**
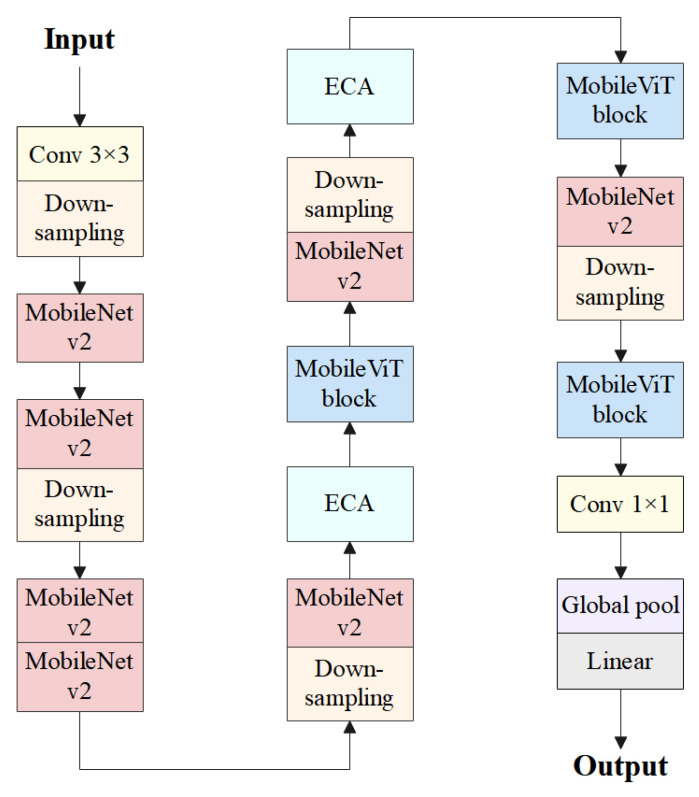
Overall structure diagram of the SFORA.

Introducing two sets of ECA modules helps enhance the model’s representational capacity in the image feature extraction stage, thereby better capturing critical local and global information in the images and thus improving the accuracy and robustness of the sheep face orientation recognition. Ultimately, the SFORA was developed, and the introduction of ECA modules enabled the SFORA to maintain low weight and high efficiency while possessing stronger feature learning capabilities, providing crucial support for sheep face orientation recognition tasks. The overall structure diagram of the SFORA is shown in Figure 6.

### 2.3. Three-Dimensional Sheep Face Reconstruction

#### 2.3.1. Collection of Full-Angle Sheep Face Images

To perform three-dimensional sheep face reconstruction, it is necessary to collect sheep face images from all angles. In this study, full-angle sheep face images were collected using a sheep face image collection device at Inner Mongolia Beiqi Technology Co., Ltd. in June 2022.

The sheep face image collection device mainly consists of a conveyor belt system. The conveyor belt system is used to stabilize the sheep’s body and assist it in moving forward. During the process of collecting sheep face images, it is crucial to ensure that the sheep remains calm and natural, as this is essential for obtaining clear and effective facial images. When the sheep’s body moves vigorously, it can lead to low-quality and blurry sheep face images being captured. Therefore, in this study, the conveyor belt system was utilized to stabilize the sheep’s body, reducing its movement and allowing for a more effective collection of sheep face images.

**Figure 7:**
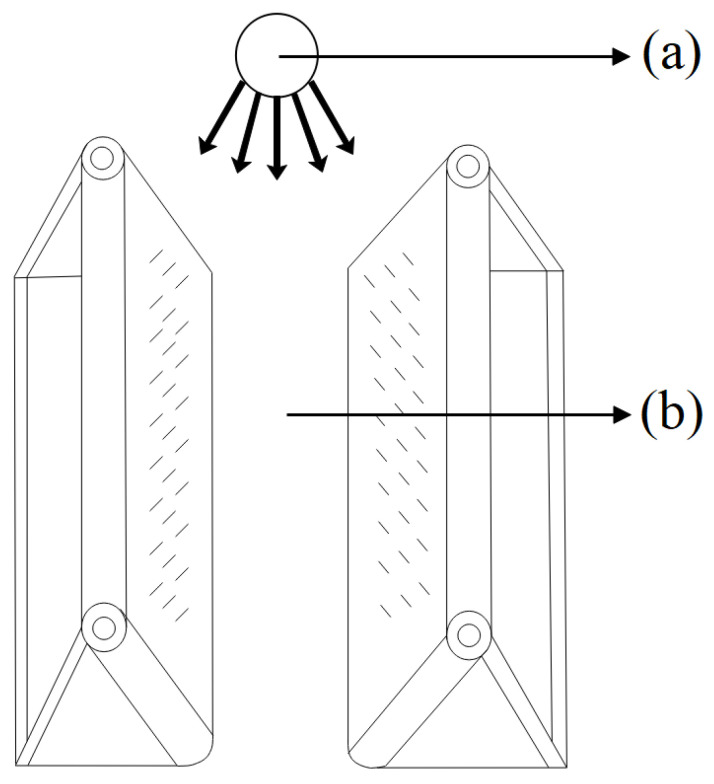
Acquisition diagram of the sheep face image acquisition device. (**a**) Shooting location; (**b**) conveyor belt system.

The conveyor belt system mainly consists of two parts: the frame structure and the clamping structure. The frame structure provides support and stability for the operation of the conveyor belt. In terms of design details, the frame structure is equipped with four rollers, each installed at the four corners of the frame structure, to facilitate the movement and adjustment of the multi-angle sheep face image collection device. The clamping structure consists of two sets of conveyor belts arranged in a “V” shape, which firmly hold the sheep’s body in place and assist it in passing through. This clamping method allows the sheep to run along a guided path, enhancing the stability of the conveyor belt system and preventing the sheep from escaping or slipping off during the image collection process. Additionally, the “V” shape structure helps prevent large movements of the sheep’s body. An acquisition diagram of the sheep face image acquisition device is shown in Figure 7.

**Figure 8:**
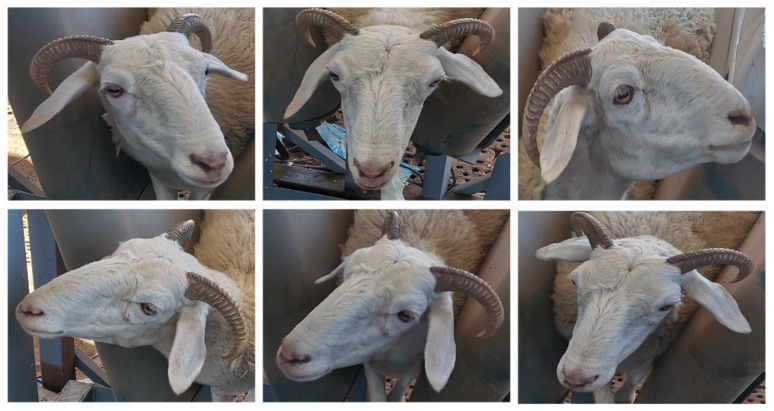
Sample images of the full-angle sheep face images collected.

The details of the full-angle sheep face image collection are as follows. When the experimental sheep pass through the conveyor belt system, the conveyor belt system is manually operated to stop. At this point, the experimental sheep are secured by two sets of conveyor belts. Subsequently, the collection personnel use the built-in camera of the Honor 9X smartphone to capture full-angle facial images of the experimental sheep. The collection took place at 3:00 p.m. A total of 10 experimental sheep were used. Sample images of the full-angle sheep face images collected are shown in Figure 8.

#### 2.3.2. Three-Dimensional Sheep Face Reconstruction

**Figure 9:**
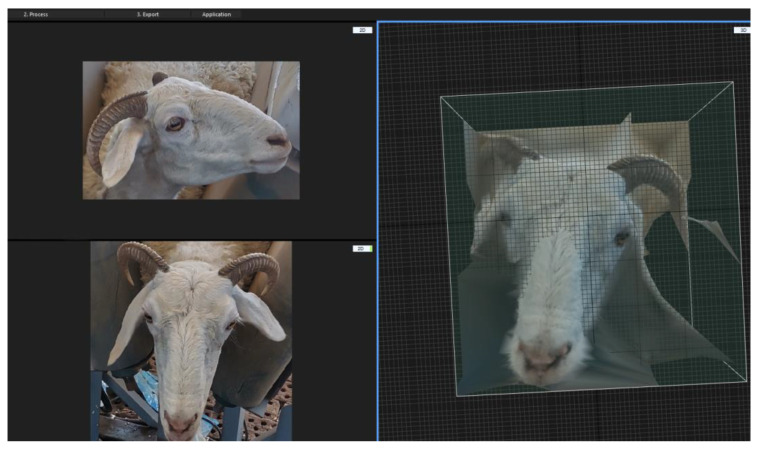
Sample images of the generated three-dimensional sheep face models.

Reality Capture is a professional three-dimensional reconstruction software that utilizes image processing and computer vision techniques to calculate spatial coordinates from a set of captured images, thereby generating high-quality three-dimensional models. In this section, the captured full-angle sheep face images are entered into this software. By processing these images, the software achieves the three-dimensional transformation of the full-angle sheep face images and generates three-dimensional sheep face models. After generating the three-dimensional sheep face models, additional textures and details are supplemented and added. The software version used in this section is Beta 1.0. Sample images of the generated three-dimensional sheep face models are shown in Figure 9.

When inputting the full-angle sheep face images into Reality Capture for three-dimensional sheep face model generation, it was found that the initially generated models were not ideal, containing a large number of background areas that affected the quality and accuracy of the three-dimensional models. To improve the effectiveness of the three-dimensional models, this section details the method of adding keypoints in the software. By manually setting keypoints in the input images, the software focused on the corresponding key areas, thereby generating three-dimensional sheep face models with better reconstruction results. Specifically, after generating the initial three-dimensional sheep face model, it is possible to specify areas with poor processing results as needed, allowing the software to reprocess based on this foundation to achieve better reconstruction results. This iterative processing method effectively improves the quality and accuracy of the three-dimensional sheep face model, making the final reconstruction results more accurate and reliable.

**Figure 10:**
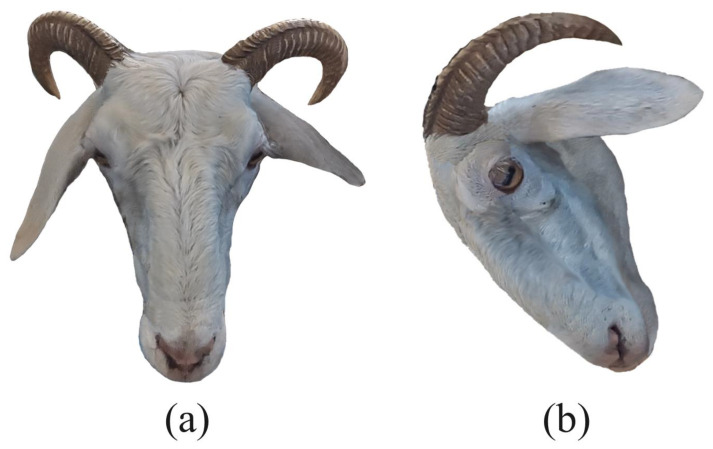
(**a**) Example of the frontal face of the three-dimensional sheep face model; (**b**) example of the right-side face of the three-dimensional sheep face model.

Using the above method, a three-dimensional sheep face model was generated for each experimental sheep, and the model’s frontal face image, left-side face image, and right-side face image were captured. The captured images contain rich three-dimensional features of the sheep’s face. An example of the frontal face of the three-dimensional sheep face model is shown in Figure 10a. The sample image of the right side of the three-dimensional sheep face model is shown in Figure 10b.

#### 2.3.3. Feature Matching Algorithm

SuperGlue is a neural network architecture used for feature matching, with the primary goal of finding correspondences between two sets of input image local features and performing matching on these correspondences [31,32]. Specifically, the core idea of the SuperGlue algorithm is to employ an end-to-end neural network structure to transform two sets of local features into a set of matching descriptors, while excluding unreliable matches during the matching process [33,34]. The main components of this algorithm include the attention graph neural network and the optimal matching layer [35]. First, the attention graph neural network takes two sets of local features as input and calculates matching descriptors through mutual communication between features. This process not only considers the positions and appearances of feature points but also integrates other contextual clues, thereby improving the accuracy and robustness of matching. Subsequently, the optimal matching layer is responsible for generating a partial assignment matrix, representing the best matching relationship between two sets of features as an optimization problem and finding the optimal matching solution.

The SuperGlue architecture consists of two main modules: the attention graph neural network and the optimal matching layer. The attention graph neural network processes the extracted feature point positions and descriptors into a feature-matching vector *f*, which is then augmented for enhanced feature-matching performance using both self-attention and cross-attention mechanisms. The specific steps are as follows: firstly, the attention graph neural network passes through a keypoint encoder, where each keypoint *i* is defined with an initial representation xi(0), combining both appearance and positional information of keypoint *i*. Subsequently, the keypoint positions are embedded into a high-dimensional vector using a multilayer perceptron (MLP), as follows:

(4) xi(0)=di+MLP(pi)

where p*_i_* denotes the position of keypoint *i* and d*_i_* denotes the visual descriptor of the keypoint *i*.

Following that, the attention graph neural network proceeds through the multiplex graph neural network, which aggregates messages on all given edges simultaneously across all nodes, computing an updated representation at each layer. The formula for updating the remaining message passing updates for all feature points *i* in image *A* is as follows:

(5) xiA(l+1)=xiA(l)+MLP([xiA(l)||mε→i])

where [·||·] denotes the connection operation and xiA(l+1) is the middle representation of the element *i* in a layer l in image *A*. The message $m_{\varepsilon \to i}$ is the result of aggregation from all keypoints $\left\{j:\right.(i,j)\in \left.\varepsilon \right\}$.

In the attentional aggregation operation, aggregation is performed through an attention mechanism to compute the message $m_{\varepsilon \to i}$. Similar to a database query where the representation of *i*, query *q_i_* retrieves values of some elements’ attributes $v_{j}$ with keys $k_{j}$. The weighted average of the above message calculations yields the message $m_{\varepsilon \to i}$, which is shown as follows:

(6) $$a_{ij}=\mathrm{Softmax}({q_{i}}^{T}k_{j})$$

(7) $$m_{\varepsilon \to i}=\sum_{j:(i,j)\in \varepsilon }a_{ij}v_{j}$$

In the optimal matching layer of SuperGlue, a matching matrix is generated, similar to a linear assignment problem, by computing a scoring matrix under all possible matches to obtain assigned keypoints and ultimately pairwise scores using the similarity of matching descriptors. Additionally, to enable the network to suppress some keypoints, a set is collected to allocate mismatched keypoints to it. Finally, keypoint scores are increased by filling in a learnable parameter.

In conclusion, the SuperGlue algorithm demonstrates efficiency and flexibility. Thanks to its end-to-end neural network structure, it can rapidly perform feature extraction and matching processes, reducing additional computational overhead and memory consumption. In this study, the SuperGlue algorithm was employed to match features between input images and cropped images of three-dimensional sheep face models. The sheep with the highest number of matches and the best matching performance were selected as the final matching result.

## 3. Results

### 3.1. Training Evaluation

In this study, the training platform consisted of an Intel Core i7-9700 CPU, an NVidia GeForce RTX 2080Ti 11 GB GPU, and 16 GB of RAM, and ran on the Windows 10 operating system. The software platform included PyCharm, with CUDA 11.3, PyTorch version 1.10.0, and Python version 3.8 installed. During model training, the dynamic initialization learning rate was set to 0.001, the number of training epochs was 50, the batch size was 64, and the approximate number of training iterations was 9000.

### 3.2. Evaluation Indicators

In this study, the evaluation metrics for the sheep face orientation recognition algorithm include accuracy, recall, precision, F1 score, model size, and mean matching time, which are used to assess the performance of the model.

Model size represents the storage space required for deploying the model. Accuracy refers to the proportion of correctly classified samples to the total number of samples. Precision indicates the percentage of correctly classified samples out of the total number of samples. Recall represents the ratio of the number of samples correctly retrieved to the total number of samples that should be retrieved. Here, TP, FP, TN, and FN stand for the number of true positives, false positives, true negatives, and false negatives, respectively. The F1 score considers both precision and recall. The ranges of precision, F1 score, recall, and accuracy are from 0% to 100%. The formulas for calculating these metrics are as follows:

(8) $$\mathrm{Accuracy}=\frac{TP+TN}{TP+TN+FP+FN}$$

(9) $$\mathrm{Precision}=\frac{TP}{TP+FP}$$

(10) $$\mathrm{Recall}=\frac{TP}{TP+FN}$$

(11) $$F1\;\mathrm{score}=\frac{2\times \mathrm{Precision}\times \mathrm{Recall}}{\mathrm{Precision}+\mathrm{Recall}}$$

In the feature-matching algorithm, this study introduced the concepts of total matching points, correct matching points, and incorrect matching points to evaluate the effectiveness of feature matching. Among these, the total number of correct and incorrect matching points equals the total number of matching points. Based on the relationship among these three, matching precision and matching recall are further calculated using the following formulas:

(12) $$\mathrm{Matching}\;\mathrm{precision}=1-\frac{\mathrm{Incorrect}\;\mathrm{matching}\;\mathrm{points}}{\mathrm{Total}\;\mathrm{matching}\;\mathrm{points}}$$

(13) $$\mathrm{Matching}\;\mathrm{recall}=\frac{\mathrm{Correct}\;\mathrm{matching}\;\mathrm{points}}{\mathrm{Correspondences}}$$

where Correspondences denotes the number of repeated matching points.

When testing the feature matching algorithm, 10 sets of reconstructed three-dimensional sheep face images and trial images were randomly selected for matching, and the average of these results was taken as the final matching result. The range of matching precision and matching recall is from 0% to 100%, with higher values indicating better feature-matching effectiveness. The mean matching time is used to test the matching speed of the feature point-matching algorithm, measured in milliseconds. The testing procedure is as follows: randomly select 10 images and input them into the feature point-matching model for matching, then calculate the average matching time.

### 3.3. Comparison with Different Recognition Models

This study utilized several classic recognition models for sheep face orientation recognition tasks and further compared the recognition results. The recognition models include YOLOv3-tiny, YOLOv4-tiny, YOLOv5s, SSD, ViT-tiny, ResNet18, and MobileViT-XXS. Each group of models was trained on the sheep face orientation dataset, and the specific training results are shown in Table 2.

From Table 2, it can be observed that MobileViT-XXS performs the best in the task of sheep face orientation recognition, with accuracy and F1 score reaching 99.0% and 99.1% respectively. Compared to the other model groups, MobileViT-XXS outperforms them with F1 score improvements of 4.7%, 3.4%, 3.0%, 2.2%, 0.4%, and 1.1%, and accuracy improvements of 5.8%, 3.7%, 3.4%, 2.0%, 0.6%, and 0.2%, respectively. Additionally, the model size of MobileViT-XXS is only 4.9 MB, which is significantly smaller compared to the other model groups, giving it a distinct advantage. In summary, MobileViT-XXS demonstrates significant advantages in both recognition accuracy and model size for the task of sheep face orientation recognition. Therefore, in this study, MobileViT-XXS was chosen as the baseline model, with the subsequent introduction of attention modules to further enhance its performance.

### 3.4. Ablation Experiment

To investigate the specific performance of the proposed improvement strategies for MobileViT-XXS in this study, ablation experiments were conducted. Specifically, MobileViT-XXS was used as the baseline model and attention modules were added to verify the model’s performance, resulting in four sets of experimental models for comparison. The first group consisted of the unimproved MobileViT-XXS model. The second group, named MobileViT-XXS-1, included an ECA module added before the first MobileViT block of MobileViT-XXS. The third group, named MobileViT-XXS-2, included an ECA module added before the second MobileViT block of MobileViT-XXS. The fourth group comprised the proposed sheep face orientation recognition algorithm, SFORA, which introduced an ECA module before the first and second MobileViT blocks. The results of the ablation experiments are shown in Table 3.

The experiments indicated that the performance of the improved models was further enhanced by introducing ECA modules according to the methods proposed in this study. Compared to MobileViT-XXS, the accuracy of the two improved models increased by 0.3% and 0.2%, respectively, after the introduction of a single ECA module, and their F1 score improved by 0.1% and 0.3%, respectively. When both sets of ECA modules were introduced simultaneously, the performance of the models was further improved. Ultimately, the proposed SFORA achieved the best performance in sheep face orientation recognition tasks, with an accuracy and F1 score of 99.6% and 99.5%, respectively. Additionally, the introduction of both sets of ECA modules increased the model size by only 0.4 MB, demonstrating a good balance between model size and recognition performance for the SFORA.

### 3.5. Performance Comparison of Different Attention Modules

This study further compared the effects of introducing different attention mechanism modules into MobileViT-XXS by conducting specific experiments. Following the improvement strategy proposed in this study, several groups of attention mechanism modules were embedded into MobileViT-XXS, with the embedding position of each group of attention mechanisms being consistent. Specifically, these include the sequence and exception (SE) module, convolutional block attention module (CBAM) module, coordinate attention (CA) module, and simple, parameter-free attention module (SimAM) module. The comparative results of different attention mechanisms are shown in Table 4.

When the SE module was introduced, compared to MobileViT-XXS, the improved model’s accuracy only increased by 0.3%, while the F1 score remained unchanged, and the model size increased by 0.4 MB. The experimental results indicate that the improvement effect of introducing the SE module is relatively poor. Additionally, after introducing the CBAM module and CA module, the accuracy and F1 score of the improved model were significantly improved, approaching the performance of the SFORA proposed in this study. However, the model size of the two improved models increased by about 1.4 MB, which is a considerable increase and therefore not adopted as the improvement solution in this study. The experimental results demonstrate that introducing the SimAM module can further improve the model’s recognition performance without increasing the model size. However, the accuracy and F1 score of the improved model are only 99.4% and 99.2%, respectively. Considering both recognition accuracy and model size, we chose to introduce the ECA module as the final improvement strategy.

### 3.6. Comparison Experiment of Confidence Thresholds for Feature-Matching Algorithms

The confidence threshold refers to a threshold used in the feature-matching process to determine whether the matching result is reliable. When the similarity score between two feature points is higher than this threshold, it is considered a reliable match; otherwise, it is deemed unreliable, possibly due to noise or erroneous matching. Adjusting the confidence threshold can affect the quantity and quality of the matching results, making the selection and evaluation of the confidence threshold crucial in feature-matching algorithms.

This study employed nine sets of confidence thresholds ranging from 0.1 to 0.9 with intervals of 0.1. Matching performance was evaluated using the matching of frontal sheep face images with the frontal views of three-dimensional sheep face models as the test object, further investigating the practical matching effectiveness of the SuperGlue algorithm. The comparative experimental results are presented in Table 5. The best matching performance was achieved when the confidence threshold was set to 0.4, with matching precision of 94.5% and matching recall of 96.0%. Conversely, the worst matching performance was observed when the confidence threshold was set to 0.7, with matching precision below 80.0%. Additionally, as the confidence threshold increased, the total number of matched points also increased. However, there was no clear correlation between the increase in the total number of matched points and the matching performance. Therefore, this study set the confidence threshold to 0.4 to further accomplish the feature-matching task.

### 3.7. Performance Analysis of Feature-Matching Algorithms

This study extracted frontal, left-side, and right-side views from the three-dimensional sheep face models for further matching tasks. We tested the matching effectiveness for the three types of face orientations, and the experimental results are shown in Table 6. When employing the SuperGlue algorithm for left-side face-matching tasks, the matching precision and recall reached 93.8% and 94.2%, respectively. For right-side face-matching tasks, the matching precision and recall reached 95.5% and 96.3%, respectively. When using the SuperGlue algorithm for frontal face-matching tasks, the matching precision and recall reached 94.5% and 96.0%, respectively. These experimental results indicate that the method proposed in this study can effectively accomplish the task of matching recognition images with three-dimensional sheep face images.

**Figure 11:**
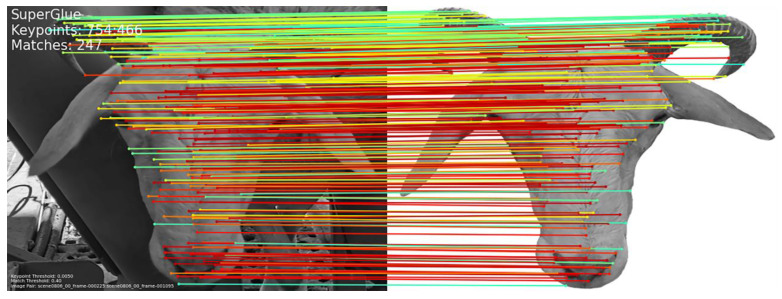
Sample image demonstrating the matching effect of a frontal face. Different feature points are matched by curves of different colors, and the matching effect is shown in Figure 11.

In terms of matching speed, the average matching times for the three types of faces were 18.8 ms, 21.9 ms, and 19.3 ms, respectively, demonstrating that the method proposed in this study has a significant advantage in matching speed. Different feature points are matched by curves of different colors, and the matching effect is shown in Figure 11.

## 4. Discussion

This study proposes a sheep identification method based on three-dimensional sheep face reconstruction and feature point matching to address the limitation of the current sheep face recognition technology’s single recognition method. Experimental results show that the accuracy and F1 score of the sheep face orientation algorithm (SFORA) proposed in this study reached 99.6% and 99.5%, respectively, with a model size of only 5.3 MB. By capturing full-view sheep face images, three types of three-dimensional sheep face images were further generated. Additionally, through SuperGlue matching experiments between three-dimensional sheep face images and target images, it was found that when the confidence threshold was set to 0.4, the model achieved the best matching performance, with matching precision for all three categories of sheep faces exceeding 93.5%.

### 4.1. Development of Sheep Identification Device

In livestock farming, the application of sheep facial recognition technology requires auxiliary devices. Specifically, sheep are timid and difficult to tame. Therefore, efficiently capturing sheep facial images for subsequent training and effectively stabilizing the sheep’s state for recognition are challenges faced by current sheep facial recognition technology. To address these issues, this study has developed a set of sheep identification devices to improve the efficiency of sheep identification and meet the practical requirements of farm applications.

The sheep identification device developed in this study is consistent with the sheep face image collection device introduced in Section 2.3.1. Similar to the sheep face collection process, maintaining a natural and calm state of the sheep is crucial during the sheep identification process and is a prerequisite for successful identification. Specifically, the identification camera needs to capture real-time facial images of the target sheep. As the sheep’s body tends to move frequently and vigorously, it can lead to low-quality and blurry identification images. Therefore, this study aims to stabilize the sheep’s body to enable the identification camera to more effectively collect facial images.

**Figure 12:**
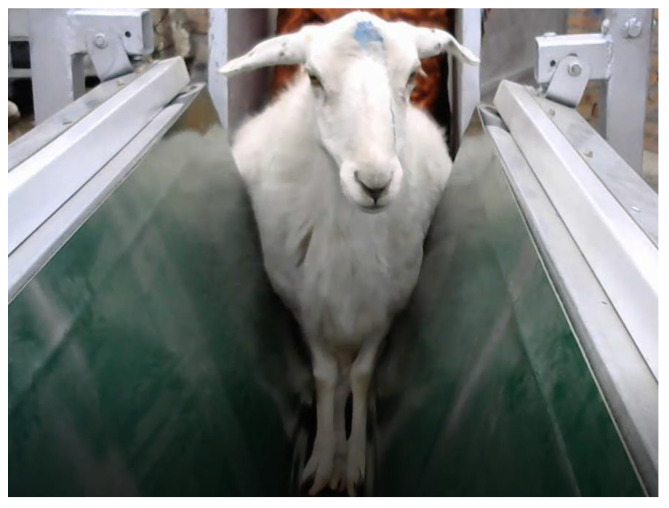
Actual operation images of the sheep identification device.

Similarly, during the initial design phase of the sheep identification device, several preliminary tests were conducted to assess its applicability and effectiveness. The tests involved 30 experimental sheep and included the following procedures. Firstly, the device was operated for three hours continuously to evaluate its stability during prolonged operation. Subsequently, the experimental sheep were individually guided through the conveyor belt system, and manual observations were made to ensure that the device did not cause any harm to the sheep. After all experimental sheep had passed through the system, technical personnel inspected the device for any deformations or damage. Additionally, veterinary professionals conducted health checks on the experimental sheep. The test results indicated that the sheep identification device designed in this study could effectively immobilize and transport the sheep rapidly without causing any harm. Actual operation images of the sheep identification device are shown in Figure 12.

### 4.2. The Application of Research Methodology in Real-World Scenarios

During the identification process, the method proposed in this study follows a unique recognition workflow outlined as follows, assuming a total population of 200 sheep on the farm. Firstly, using three-dimensional reconstruction technology, the face of each experimental sheep is modeled in three dimensions, further obtaining a three-dimensional sheep face model. Secondly, the front, left, and right profiles of the three-dimensional sheep face model are manually captured and stored in the farm server’s database.

When performing identity recognition, the recognition camera captures real-time facial images of the target for recognition. The captured recognition images are first processed by the sheep face orientation recognition algorithm proposed in this study to determine the orientation, which includes front, left, and right orientations. After determining the orientation of the recognition image, it is matched with the corresponding three-dimensional facial images of all sheep in the database using a feature-matching algorithm. The identity information of the recognition target is obtained from the output with the highest matching result. These operations efficiently and quickly complete the entire recognition task.

Currently, there are relatively few recognition methods for sheep face technology. In the future, more recognition methods need to be evaluated and developed. At the same time, the use of deep learning technology for three-dimensional sheep face reconstruction holds significant research significance. In the long run, developing a model specifically for sheep face feature point matching would be beneficial. Ultimately, the developed sheep face image collection device and sheep identification device should be deployed in more breeding farms, becoming part of digitized and intelligent breeding farms that integrate big data analysis.

## 5. Conclusions

This study applied three-dimensional sheep face reconstruction technology to the task of sheep identification and further developed a sheep face orientation recognition algorithm and a feature point-matching model using deep learning techniques, achieving efficient matching of sheep identities. Through the above methods, the aim was to address the issue of limitations in sheep face recognition technology. Ultimately, the proposed sheep face orientation recognition algorithm, SFORA, achieved accuracy and F1 score of 99.6% and 99.5%, respectively, with a model size of 5.3 MB. Additionally, when the confidence threshold was set to 0.4, the matching performance of the feature-matching model was optimal, with matching accuracies of 96.0%, 94.2%, and 96.3% for sheep’s frontal, left-side, and right-side faces, respectively. Experimental results demonstrate that the proposed method achieves high accuracy and effectively completes sheep face recognition tasks. Through a non-invasive identification approach, potential harm to the sheep is avoided. This method not only achieves sheep identification but also meets the requirements of modern digital agriculture practices in combining artificial intelligence, big data, and remote automation. This study enriches the theoretical research on sheep face recognition and provides technical support for its practical application.

In this study, three-dimensional images of the faces of 10 experimental sheep were generated using three-dimensional reconstruction software. In future research, it would be beneficial to develop a model specifically for three-dimensional sheep face reconstruction and feature point matching based on deep learning techniques. Additionally, it is necessary to increase the number of experimental sheep and consider the impact of environmental factors on the recognition performance.

## Figures and Tables

**Table 1:** The specific configuration of the sheep face orientation dataset.

Dataset	Images	Proportion	Number of Experimental Sheep
Training	11,520	80%	30
Validation	1440	10%	
Testing	1440	10%	

**Table 2:** Summary of results of different recognition models.

Model	Accuracy (%)	F1 Score (%)	Model Size (MB)
YOLOv3-tiny	93.2	94.4	33.7
YOLOv4-tiny	95.3	95.7	22.6
SSD	95.6	96.1	99.5
YOLOv5s	97.0	96.9	14.0
ResNet18	98.4	98.7	44.7
ViT-tiny	98.8	98.0	22.5
MobileViT-XXS	99.0	99.1	4.9

**Table 3:** Summary of results of the ablation experiment.

Model	Accuracy (%)	F1 Score (%)	Model Size (MB)
MobileViT-XXS	99.0	99.1	4.9
MobileViT-XXS-1	99.3	99.2	5.1
MobileViT-XXS-2	99.2	99.4	5.1
SFORA	99.6	99.5	5.3

**Table 4:** Summary of results of introducing different attention modules.

Model	Accuracy (%)	F1 Score (%)	Model Size (MB)
MobileViT-XXS	99.0	99.1	4.9
MobileViT-XXS + SE	99.3	99.1	5.3
MobileViT-XXS + CBAM	99.6	99.4	6.3
MobileViT-XXS + CA	99.5	99.5	6.2
MobileViT-XXS + SimAM	99.4	99.2	4.9
SFORA	99.6	99.5	5.3

**Table 5:** Experimental results with different confidence thresholds.

Model	Confidence Threshold	Matching Precision (%)	Matching Recall (%)	Total Matching Points
SuperGlue	0.1	84.3	86.9	290
	0.2	90.1	92.4	278
	0.3	93.4	92.8	262
	0.4	94.5	96.0	247
	0.5	89.1	84.6	210
	0.6	86.7	85.9	178
	0.7	78.4	85.3	153
	0.8	84.0	82.3	123
	0.9	83.9	85.5	85

**Table 6:** The training results of the feature matching algorithm.

Model	Category	Matching Precision (%)	Matching Recall (%)	Mean Matching Time (ms)
SuperGlue	Left-side face	93.8	94.2	18.8
	Frontal face	94.5	96.0	21.9
	Right-side face	95.5	96.3	19.3

## Data Availability

The dataset can be obtained by contacting the corresponding author.
